# Participant perspectives on a multimodal program for neuropathic pain after spinal cord injury

**DOI:** 10.3389/fpain.2026.1755081

**Published:** 2026-02-13

**Authors:** Meredith Pinkerton, Roberta Vastano, Kim D. Anderson, Marlon Wong, Gabriel Fernandez, Eva Widerström-Noga

**Affiliations:** 1 The Miami Project to Cure Paralysis, University of Miami, Miami, FL, United States; 2 Neuroscience Graduate Program, University of Miami, Miami, FL, United States; 3 Department of Neurological Surgery, University of Miami, Miami, FL, United States; 4 Department of Physical Medicine and Rehabilitation, MetroHealth System, Case Western Reserve University School of Medicine, Cleveland, OH, United States; 5 Department of Physical Therapy, Miller School of Medicine, University of Miami, Coral Gables, FL, United States

**Keywords:** bodily illusions, exercise, neuropathic pain, nonpharmacologic approach, pain programs, spinal cord injury

## Abstract

**Introduction:**

Spinal cord injury (SCI) results in several medical consequences. While some of these are immediate, others, like chronic pain, may emerge after weeks or months. Roughly 60% of individuals develop persistent neuropathic pain within the first year after injury. Due to the limited efficacy and negative side-effects of current pharmacological agents, many patients seek non-pharmacological options.

**Methods:**

The present mixed-method study explored individual perspectives of 35 participants with neuropathic pain and SCI on a multimodal 12-week pain program including pain education, exercise, and walking illusions.

**Results:**

After completion of the program participants reported reduced pain and pain interference, better understanding of pain and pain management, and lower medication use. Other reported benefits included an expanded mindset, useful community and interpersonal connections, positive health impact, increased motivation, and elevated self-image. Negative perceptions included fatigue, short-lasting or minimal effects on pain, and lack of realism of walking illusions. Pain assessment scores supported the overall positive effects on neuropathic pain with significant reductions in neuropathic pain severity, pain interference with activities and mood, and difficulty dealing with pain. These results suggest that a multimodal pain program combining pain education, exercise and walking illusions can reduce neuropathic pain and its impact after SCI.

## 1 Introduction

Among the numerous consequences of spinal cord injury (SCI), about 80% of individuals develop persistent pain within 12-months post injury (1), with 60% experiencing neuropathic pain (1). The refractory nature of neuropathic pain often significantly and negatively impacts quality of life (QOL) after injury (2). Pharmacological standard treatments include anti-convulsants (i.e., pregabalin and gabapentin) and tricyclic anti-depressants (i.e., amitriptyline) (3). However, these medications do not adequately alleviate pain for everyone (4), have numerous adverse side effects (5), and can be misused (6). Therefore, many people who experience neuropathic pain would like to have greater access to non-pharmacological options to better manage their pain (7). Non-pharmacological interventions used in SCI chronic pain research studies have for example included transcranial magnetic stimulation (TMS) (8–10), transcutaneous electrical nerve stimulation (TENS) (11, 12), transcranial direct current stimulation (tDCS) (13, 14), walking illusions, alone (15) and in combination with tDCS (16).

However, many of these interventions have been used in isolation and it is well known that chronic pain is a multidimensional experience influenced by multiple factors (17); therefore, a combination of treatments to manage SCI-related neuropathic pain may be optimal (7). One approach that can be easily incorporated with other treatments options is pain education. Indeed, patient education regarding pain following SCI has previously successfully been incorporated with both pharmacological and non-pharmacological interventions, and has been shown to improve pain outcomes (18). Pain education for chronic pain in SCI should include information regarding the underlying neurophysiological mechanisms of pain and be based on participant feedback regarding content (18, 19).

Similarly, other studies utilizing bodily illusions, like walking illusions (15, 20, 21), have shown that the manipulation of multisensory inputs like visual, tactile, and proprioceptive information can modulate body representation and potentially reduce neuropathic pain (22). After SCI, the deficits in somatosensory and motor function (23) have been linked to altered body perceptions and deficits in body representation in this population (22, 24–27). The analgesic effects of bodily illusions is caused by a correction of sensory-motor mismatch (15), activation of the somatosensory cortex (21), and modulation to other cortical regions when combined with tDCS (20). Although interesting, these types of studies mainly focused on mechanisms that generate analgesic effects and to date participants' perspectives on this type of intervention are still missing.

Finally, exercise in different forms has been shown to be potentially powerful for reducing the severity and negative impact of neuropathic pain. Although there is little research on the effects of exercise on SCI related neuropathic pain, there is compelling evidence that exercise improves pain outcomes in people with peripheral neuropathic pain (28, 29), heterogeneous central neuropathic pain (30), and heterogenous chronic pain (31, 32). There are numerous processes that may be underlying the analgesic effects of exercises (33) including neuroimmune mechanisms (31, 34, 35), modulation of inflammation (36, 37), and activity-dependent neuronal plasticity (36). Transient exercise-induced hypoalgesia has also been demonstrated within a single bout of exercise (38, 39) and has been shown to decrease pain sensation and temporal summation (40, 41). Therefore, the analgesic effects of exercise may partly be due to modulation of neuronal hyperactivity. Neuropathic pain is also closely linked to psychosocial factors, such as life interference, and exercise has improved overall wellness, cardiometabolic risk profile (42–44), psychosocial risk (45, 46) and functional ability (31, 47). However, despite these benefits, perspectives of individuals with SCI on exercise interventions remain largely unexplored.

Understanding participants' view on educational resources, bodily illusions and exercise components is crucial for designing patient-centered programs. Incorporating this feedback can help tailor non-pharmacological approaches alongside other pain management strategies. Therefore, with the repeated requests from the SCI community to have better access to non-pharmacological treatments options (48) and the emergence of promising multimodal approaches to make neuropathic pain manageable (49), we designed this mixed-method study to gain insights into participants' perspectives and explore neuropathic pain outcomes after a 12-week program including pain education, walking illusions and exercise sessions. The present study aimed to significantly expand on previous research in this population (50) by providing detailed feedback on the pain program by study participants with SCI who experienced moderate to severe neuropathic pain. Additionally, the present study evaluated pain and psychosocial outcome scores to complement the participant perspectives.

## 2 Methods

### 2.1 Study participants

Study participants were recruited via flyers posted at the Miller School of Medicine and from The Miami Project's research volunteer database. The study adhered to the principles of the Declaration of Helsinki. The study was reviewed and approved by Institutional Review Board of the University of Miami Miller School of Medicine and the Department of Defense Office of Human and Animal Research Oversight. All participants went through a screening and an informed consent process and provided written consent after confirmed eligibility.

**Figure 1:**
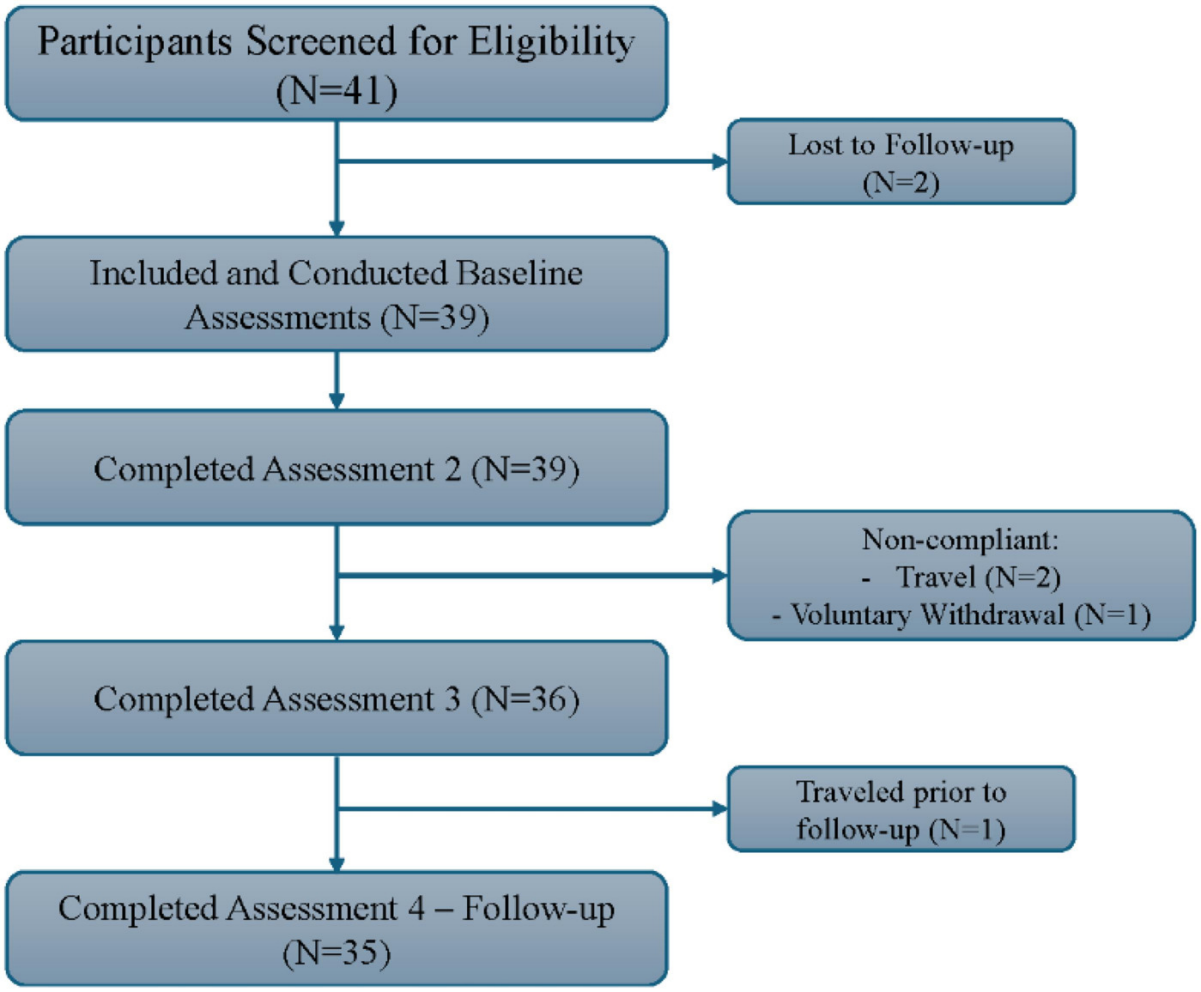
Flowchart of participant's retention.

Potential participants with a history of systemic illness (e.g., cardiovascular disease, multiple sclerosis, rheumatoid arthritis, cancer), severe depression (BDI-II > 29) (51), body mass index (BMI) > 35 (52), unhealthy alcohol (AUDIT>10) (53) or drug (DAST-10 > 6) (54) use within the past year, were not eligible for the study. Thirty-five adult participants with SCI completed the study (Figure 1). Participants included both men and women, aged 19–74 years, with either complete or incomplete traumatic SCI (C2-T12) who had experienced moderate to severe neuropathic pain for a minimum of 6 months [numeric rating scale (NRS) ≥ 4/10]. Participant demographics and injury characteristics are displayed in Table 1.

**Table 1:** Demographic information.

Participant	Age	Sex	Time Since Injury (Years)	ISNCSCI	Injury Level	Cause of Injury
1	30	F	2	D	C4	Fall
2	74	M	12	D	C2	Fall
3	51	M	4	D	T1	Act of violence
4	45	M	16	D	C1	Vehicle accident
5	19	F	5	D	C4	Vehicle accident
6	61	M	39	A	C5	Vehicle accident
7	62	M	18	D	C5	Fall
8	34	F	10	C	T10	Vehicle accident
9	69	F	1	A	T3	Sporting accident
10	19	M	0.5	–^a^	T11-T12	Act of violence
11	31	M	11	C	C4	Vehicle accident
12	33	M	9	A	C2	Vehicle accident
13	35	M	4	B	C4	Fall
14	38	F	4	A	T12	Vehicle accident
15	26	F	1	B	L1	Fall
16	41	M	2	B	C4	Vehicle accident
17	39	M	6	A	T10	Vehicle accident
18	40	F	23	A	C8	Vehicle accident
19	21	M	2.25	A	C6	Sporting accident
20	47	M	23, 3	D	L3-S1, C3-C6	Other cause
21	43	M	5	D	T10	Fall
22	37	F	12	D	T5	Vehicle accident
23	43	M	8	D	C8	Act of violence
24	50	M	27	D	C6	Vehicle accident
25	57	M	11	D	T7-T9	Other cause
26	53	M	30	C	C5	Other cause
27	43	M	8	C	C5	Fall
28	54	M	6	D	C4	Vehicle accident
29	55	M	8	A	C4	Vehicle accident
30	36	M	1.25	B	C7	Vehicle accident
31	30	M	6	C	T10	Vehicle accident
32	28	M	7	A	T5	Vehicle accident
33	40	M	0.92	A	T5	Vehicle accident
34	42	M	6	A	C6	Sporting accident
35	29	M	9	A	T12	Fall

M, Male; F, Female; C, cervical; T, thoracic; L, lumbar; S, sacral. ISNCSCI, International standard for neurological classification of SCI.

a Completeness of injury was not determined.

### 2.2 Pain program design

This manuscript includes a subset of data from a larger 12-week mixed-methods research study. The overall pain program included four weekly 1-h group sessions of pain education (19), followed by six weeks of biweekly upper body exercise incorporating walking illusion, and concluded with a four-week follow-up period, shown in Table 2. This manuscript focuses on the qualitative and quantitative outcomes following the exercise and walking illusion interventions and the follow-up assessments as the results after pain education component were published elsewhere (55). The details of the pain program and its development were described in our previous articles (55, 56).

**Table 2:** Study timeline with assessments given following each activity block.

Assessment 1	Assessment 2 (Week 5)	Assessment 3 (Week 11)	Assessment 4
Screening	Pain Education	Exercise and Bodily Illusion	Follow-up
- Demographic and Injury	- Pain Outcomes	- Pain Outcomes	- Pain Outcomes
Characteristics	- Sensory Outcomes	- Sensory Outcomes	- Sensory Outcomes
- Pain Outcomes	- Psychosocial Outcomes	- Psychosocial Outcomes	- Psychosocial Outcomes
- Sensory Outcomes	- Qualitative Interview	- Qualitative Interview	- Qualitative Interview
- Psychosocial Outcomes			

#### 2.2.1 Pain education

During weeks 1–5, all participants completed a pain education block. Educational material was presented in a multitude of ways, including written and oral presentations and guided group discussions. All learning material was taken from the educational resource, *SeePain* (19). The material presented was regarding: (1) Types of Pain, Pain Experience, and Pain Mechanisms; (2) Real-life Cases of Pain after SCI; (3) Chronicity and Meaning of Pain after SCI; (4) Impact on Life; (5) Approaches to Managing Pain (pain medication, non-pharmacological treatments, self-management, and coping strategies); and (6) Literature resources and web links. The intent of pain education was to improve the participants' understanding of causes and types of pain and how to accurately communicate about their pain to family members and the healthcare community. Also, the pain education was aimed to clearly inform participants what to expect for long-term pain, how to access information regarding pain, and understanding pain management options. Data and details concerning the pain education sessions have already been published (57).

#### 2.2.2 Exercise and walking illusion

**Figure 2:**
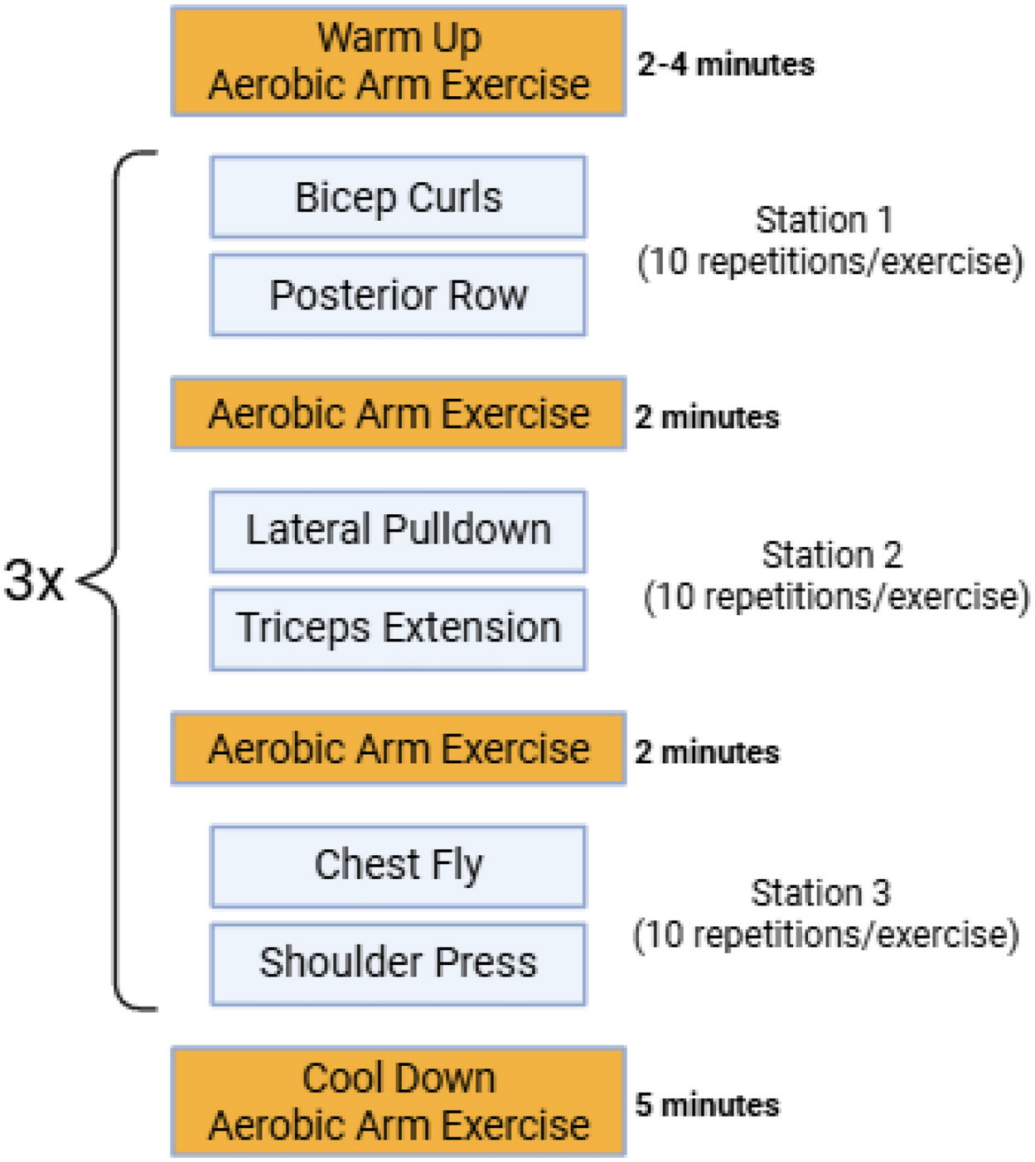
Schematic of training circuit.

Following pain education, individuals participated in 6-week biweekly upper body exercise circuit and walking illusion sessions. Biweekly sessions were administered individually to each participant. The type and duration of exercise were designed based on previous literature on exercise programs for SCI populations (54, 57, 58). All exercises were performed under the supervision of trained staff and investigators. Due to injury level or increased pain severity, adaptations were provided if needed. The resistance circuit, shown in Figure 2 consisted of bicep curls, posterior row, lateral pulldown, triceps extension, chest fly, shoulder press, and an aerobic arm exercise (arm bike or arm ergometer). Each exercise was performed with machines, resistance bands, and/or dumbbells. Movements were conducted to the subject's full range of motion and subjects were encouraged to maintain the same load. The training session began with a 2–4-min aerobic arm exercise warm-up and concluded with a 5-minute aerobic arm exercise cool down. The circuit consisted of 3 stations with 2 exercises in each station, separated with a 2-min aerobic arm exercise. Each exercise included 10 repetitions, with one repetition being 6-s total (3-s concentric, 3-s eccentric). The circuit was completed 3 times with a 15 s rest between stations, which brought the entire training program duration to 45 min. During the exercise sessions, momentary pain was recorded following each exercise. Blood pressure was measured before and after exercise for safety reasons. At the end of each circuit, exercise intensity was assessed using the Borg Rating of Perceived Excretion Scale (59).

Following each exercise session, participants underwent a walking illusion. The walking illusion session was conducted as previously shown (15). Participants were seated in their wheelchairs facing a half-mirror, with their upper bodies visible in the mirror and their lower bodies hidden by a black blanket. A 20-min video was projected beneath the mirror, showing the legs of a person walking on a treadmill. The video was tailored to the participant's sex: men viewed males' legs and women viewed female legs. Participants were instructed to move their upper bodies in synchrony with the walking motion in the video, creating a visual experience that they were watching themselves walk.

### 2.3 Questionnaires and pain assessments

#### 2.3.1 Pain evaluations

Pain evaluations were conducted in interview formats at each assessment week (baseline, 5, 11, and 15).

##### 2.3.1.1 Pain history and classification

The International SCI Pain Basic Dataset (60) is part of the NIH SCI Common Data Elements and was used to provide a pain classification and overall description of all pain experienced. In addition, we used the Appendix A of the International SCI Pain Extended Dataset (60) which includes 7 questions regarding temporal pattern of pain, worst pain intensity, average pain unpleasantness, number of days with manageable pain, and momentary pain intensity.

##### 2.3.1.2 Neuropathic pain severity

The Neuropathic Pain Symptom Inventory (NPSI) is a 12-item questionnaire, which was used to assess the severity of common neuropathic pain symptoms (61). The NPSI assesses five different dimensions of neuropathic pain: burning pain, pressing pain, paroxysmal pain, evoked pain, and paresthesia/dysesthesia. The NPSI has adequate psychometric properties in people with SCI (62).

#### 2.3.2 Psychosocial evaluations

We used the SCI version of the Multidimensional Pain Inventory, MPI-SCI, to assess the psychosocial impact of pain (63). This was adapted from the West Haven-Yale Multidimensional Pain Inventory (MPI) which is a comprehensive instrument designed to assess a range of self-reported behavioral and psychosocial factors associated with chronic pain symptoms (63). The MPI-SCI consists of 50 items, that are answered on a 7-point Likert scale. In this manuscript, pain severity was assessed via the MPI subscale.

### 2.4 Qualitive interviews

Interviews were conducted via Zoom using an interview guide to ensure consistency. The interview guide probed the individual perspectives on the pain program. The interviews also examined the perceived impact of pain education, exercise, and walking illusion on participants' ability to manage their pain. During each interview, participants were asked a set of predetermined open-ended questions:

(i) Manageable pain: Please describe what manageable pain means to you right now.
(ii) Please describe your experience with the pain education/exercise/visual illusion part of the study.
  (a) What did you like about it?
  (b) What did you not like about it?
  (c) What were the benefits, if any, of pain education/ exercise/visual illusion?
  (d) What were the risks or burden, if any, of pain education/ exercise/visual illusion?
(iii) What impact, if any, did it have on your ability to manage your pain?

Qualitative data is influenced by multiple factors that can affect responses in several ways, and therefore this type of data should not be treated as discrete, independent responses to be counted. Due to the open-ended qualitative interview questions, systematically quantifying each theme could potentially bias the qualitative data as some participants may not spontaneously express a specific opinion (64). We allowed participants to take the questions posed in the interview guide in their preferred direction, only adding probing questions related to the topic they brought up for clarification. Thus, it is not possible to ascertain whether a participant agreed or not with a specific theme if they did not mention it. The purpose of the interview probes was to gain clarity, detail, and more descriptive thoughts or feelings when necessary. To maintain a non-biased interview, we did not utilize responses from other participants to guide the conversation towards a specific theme or topic. For the purposes of the present paper only interviews conducted after the exercise/walking illusion were analyzed as their perspectives on the pain education were previously published (56).

### 2.5 Statistical plan

#### 2.5.1 Qualitative

The sample size of 35 was consistent with our previous experience in qualitative research and on recommendations for qualitative research (65). The recommendations for grounded theory qualitative studies (66) suggested that such studies should generally include between 20 and 30 interviews. We conducted 35 interviews via zoom for each of the 4 test weeks. The transcripts of the interviews were entered into NVIVO (software headquartered in Lumivero, Denver, CO), coded independently by two coders, and further discussed in team meetings to ensure credible data and introduce revisions if appropriate.

#### 2.5.2 Quantitative

Although this mixed method study was adequately powered for qualitative research the analyses of the pain and psychosocial scores were conducted to complement the qualitative analyses without statistical power considerations. Quantitative analyses were assessed for normality and homogeneity of variance using Shapiro–Wilk tests which indicated non-normality. Median scores for ISCIBPD-2 worst pain intensity, and pain interference with activities, mood, and sleep, difficulty dealing with pain and MPI sub-scales (pain severity, life interference, life control, affective distress, support, general activity, and pain impacting general activity), and total intensity NPSI score, were compared between baseline and post-pain education (week 5), post-exercise and bodily illusion (week 11), and post-follow-up (week 15). Non-parametric Friedman's test was used and pairwise *post-hoc* comparisons were uncorrected (Dunn's). All statistical analyses were conducted using GraphPad Prism version 10.0.0 for Windows, GraphPad Software, Boston, Massachusetts USA. Results were considered significant if values met *p* ≤ 0.05.

## 3 Results

**Figure 3:**
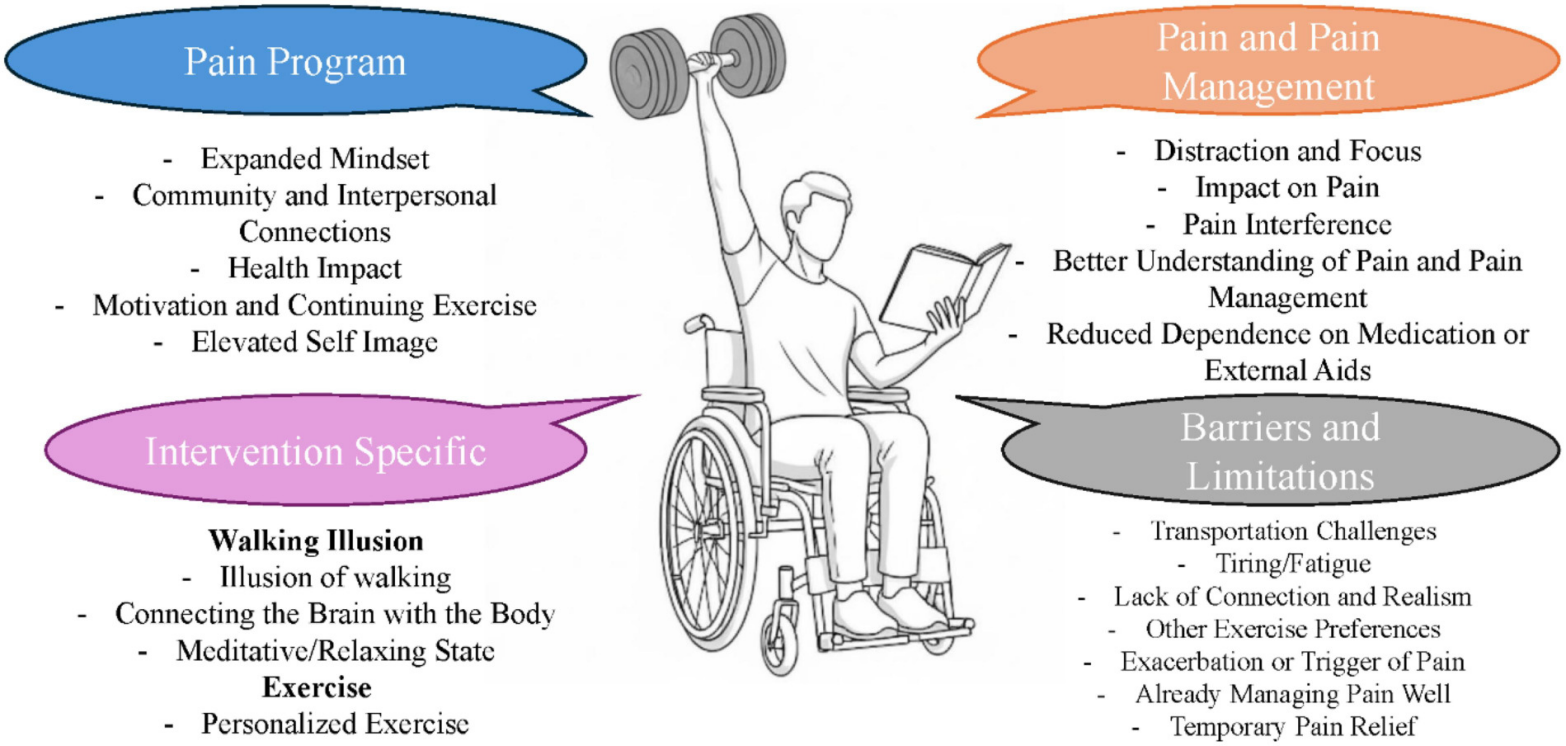
Schematic of overarching themes and subthemes based on qualitative interviews of participant's perceptions on the pain program.

The qualitative interviews reflected participants' perspectives directly after completing the pain program and at a follow-up, which was 4 weeks later. The main themes that were identified with representative quotes are summarized below. Participant perspectives with respect to benefits were categorized into the following over-arching themes regarding: (1) The pain program overall, (2) The specific interventions, (3) Pain and pain management, and (4) Barriers and limitations. Within these topics, specific subthemes were found and are listed in more detail below and in Figure 3.

### 3.1 Pain program

#### 3.1.1 Expanded mindset

Following the pain program, several participants stated that this program had influenced how they viewed their lives, “*I can have a better life than what I'm living. There's more out there than I'd realized.”* Some stated that it was “*like a mindset shift of what I could accomplish in life now that I'm in a wheelchair”*. Participants mentioned that they felt “*not as constricted by pain”* and instead of “*just laying in bed*,*”* some held the view that “*you're better off going, even though you're in pain, just take your meds*.*”* This program impacted participants differently; however, a common perspective on the benefits of the pain program shared by many participants is illustrated by “*I'm processing pain differently.”*

#### 3.1.2 Community and interpersonal connections

Some participants mentioned that they valued being part of a supportive community and not being alone reflected in one participant's quote “*I'm not alone in this*,”. Another participant appreciated the sense of community, saying that it's “*hard to get a group of guys that's had spinal cord injury*”. Participants felt that “*the interaction was a big part”*, with some sharing the benefit of, *“being able to be around the other people that are like me and talk to them and figure out things, and they tell me things that I don't know.”* and the “*wealth of knowledge amongst, not only the staff… but also stuff like spending time with other people in wheelchairs”*. Many participants shared similar feelings, with one expressing, “*the value of that there, there's no price I could put on that”.* These perspectives highlight the value of community interaction in addition to specific interventions.

#### 3.1.3 Health impact

Participants reported improvements in strength, mobility, and overall physical health. Many participants reported improvements in upper body strength *“I'm not dragging myself,”, “I really see how, day to day, that upper body strength is really helping me”,* and *“Now I do believe my upper body is stronger, and I think because of that also, I might've clicked in better posture”.* Some participants also noticed the benefit of increased core strength, illustrated by “*my core body has gotten stronger so I'm not falling over*” and “*I could shift and I could stay there in a better position for a while”*. Due to the increase in strength one participant noticed, *“I can react faster, you know, stop or dodge something in the way”*. Several participants attributed reduction in pain to be a result of the exercise, with one participant saying that the program “*Increased my strength and decreased my pain”*, and that “*the more I exercise it out, the less tight that knot becomes*,*”* and that their muscles are “*not stiff*” after exercise. Participants also noted other health benefits, “*My blood pressure's been coming down”* and that prior to the study, “*my cholesterol was slightly elevated… I was able to lower it 50 points”* by the 15-week follow-up. Others reported that “*I mean, it made my life easier, it makes me stronger”* and that “*I start the day in another mood”.*

#### 3.1.4 Motivation and continuing exercise

Participants were encouraged by their progress throughout the program and got motivated, many of them stated *“I didn't even know that I could do all that so I'm super happy and really motivated”,* Additionally, some participants described feeling motivated also outside the research environment *“I have more motivation to do everything. So I'm so happy”* and *“I'm always constantly moving. And then, my pain, I don't think of my pain. It really takes my pain away.”*

Many participants were encouraged to incorporate what they learned during the program, saying “*pushed me to start using the arm cycle again”, “I've been working out every day, trying to, and just trying everything that I've learned” “Like I'm going to continue working out on my own.”.* With many having the determination*, “I don't care about the pain. I might as well just going to face it, little by little.” Because if I keep saying, “I'm not going to do shoulders because of the pain,” then “I'll never get there.”* and to “*keep trying. Not to give up.”*

#### 3.1.5 Elevated self-image

Some participants expressed a new sense of self during this program, mentioning that they *“liked how it made me feel.”* With some expressing that “*it brings back life almost”* and “*It made me feel energized. It made me feel alive.”* Before this program, “*I didn't even know that I could work out so hard”,* and that this program is *“a little bit more challenging, which is good”.* Some participants started to “*expand the ideas of how can I work out or what can I do”* and gave the confidence to say, “*Hey, I can do these workouts. I can do them every day. I just need to do it a little different,” “it opened my mind up to trying out other equipment.”* This confidence translated in different ways for participants, with some saying, “*I'm glad I did it because there's a way to face your fears,”* and that they, “*just felt more energy again”.*

### 3.2 Intervention specific

#### 3.2.1 Walking illusion

##### 3.2.1.1 Illusion of walking

Participants felt that the walking illusion closely mimicked the sensation of walking*, “it's actually like a treadmill and it's moving, and it moves fast and it moves slow, and I find myself feeling like I'm actually moving my feet. I feel the circulation is more in my legs when I'm doing it, and so it feels like I'm really walking, and it feels good”.* Participants experienced ownership with the projected legs and reported “*seeing myself walk*” and feeling genuine movement through “*counting the steps, movements, and rapid steps, slow steps*,” creating a comprehensive sense of embodied movement. They also expressed feelings of agency (i.e., the ability to feel control over movements) “*the brain sending my body the signal, I'm walking*” and “*where you can feel yourself telling your leg to move.”* Additionally, participants actively engaged in imagination practices where they would “*pretend like I'm walking with it*” and “*as far as just trying to imagine even yourself walking again”*. A motivational aspect of this mental practice was also noted “*motivated me and it helped me imagine myself if I was walking or if I was to walk*”. Participants extended these imagination practices beyond their sessions, with some describing how they would sit “*under the tree*” and imagine “*walking around the block*” or observing people running around them and “*thinking about that*”. These experiences also had mood benefits where imagining “*I would imagine myself walking again and then walking on the beach I used to. So I would see that and I'm like, okay. It would put me in a good mood.”*

##### 3.2.1.2 Connecting the brain with the body

Participants reported that the illusion facilitated bodily reconnection and integration “*I could connect, that I was part of my body” “That kind of connects with my injury.”,* and again *“that the brain is connected”.* This also elicited positive affective responses “*I walk away from it in a very pleasant way, instead of being burdened by it”.*

##### 3.2.1.3 Meditative/relaxing state

Participants experienced meditative state and mental engagement during the bodily illusion interventions. The quotes reveal that the experience was “*almost like a meditative state*” and induced feelings of relaxation “*getting into the character they put in front of you. And I don't know, it helps out. It relaxes you” “To me, it was a relaxation type of thing.”*

#### 3.2.2 Exercise

##### 3.2.2.1 Personalized exercise

Participants reported that exercise programs were adapted to their physical limitations (e.g., weight adjustments, pacing), “*I had to use the lowest weight. If I use heavier, try to prove something, I get in trouble with my neck, my shoulder, or something like that. So the weight, they accommodated the weight for me that I could handle”* and “*we took it easy and ramped it up slowly*” with “*enough time to rest my muscles in between*,”*.* Supervision and expertise were seen as critical for maintaining safety, correcting posture, and encouraging appropriate challenges. This supportive environment encouraged participants to safely challenge themselves beyond their comfort zones, with the guidance allowing them “*to challenge myself, to do something I wouldn't normally do”* while maintaining confidence that “*with their supervision, I felt good about it*.”

### 3.3 Pain and pain management

#### 3.3.1 Benefits and facilitators

##### 3.3.1.1 Distraction & focus

Many participants perceived that the interventions “*really distracted me… pain didn't get in the way*” and “*Took my mind off the pain”.* Throughout the pain program, a new way to manage pain emerged for many participants. During the exercise intervention, participants noted that “*when you just go through and through and just keep doing it, you don't think about the pain*”; “*it reinforced me that when I exercise I can take the pain and put it somewhere else. I can exercise and don't think about my pain problem.”,* and that the bodily illusion allowed one participant to think, “*Hey look, I can take my mind from here and visualize something else to keep your mind from the pain”*. One participant expressed that this momentary relief of pain was positive because, “*being able to live without pain, even for a few hours is better than living with pain all the time*”.

##### 3.3.1.2 Impact on pain

(a) Qualitative

Most participants described how the pain program positively impacted their overall pain. When asked to rate their pain, one participant noted pain levels dropping “*from eight it went down to four, and from four sometimes I don't even feel it*” and chronic pain that “*used to always hurt would be at a constant six, maybe even a seven all day long. Right now, it's at a zero.”* Participants described both immediate relief during exercise sessions where they became “*pretty much pain-free*” and some reported specific pains like, “*shooting pain that I had in my biceps*” and “*neuropathic pain*” that's “*gotten better*.” Throughout the exercise intervention, participants noticed progressive relief, saying their pain was decreasing during the exercise sessions “*sometimes I will get there, and it's six or seven and by the time I start working they say”, “What's your pain?” I say, “I'm about four right now.” Then keep going, I said “I'm three”*. Other participants reported longer-term changes post exercise sessions, where they say, “*don't have any pain anymore and I believe it's because of the exercise that I did”*; “*I have less pain because I haven't noticed a lot of pain like before”* and “*just not in pain a whole lot now*”.

(b) Quantitative

**Figure 4:**
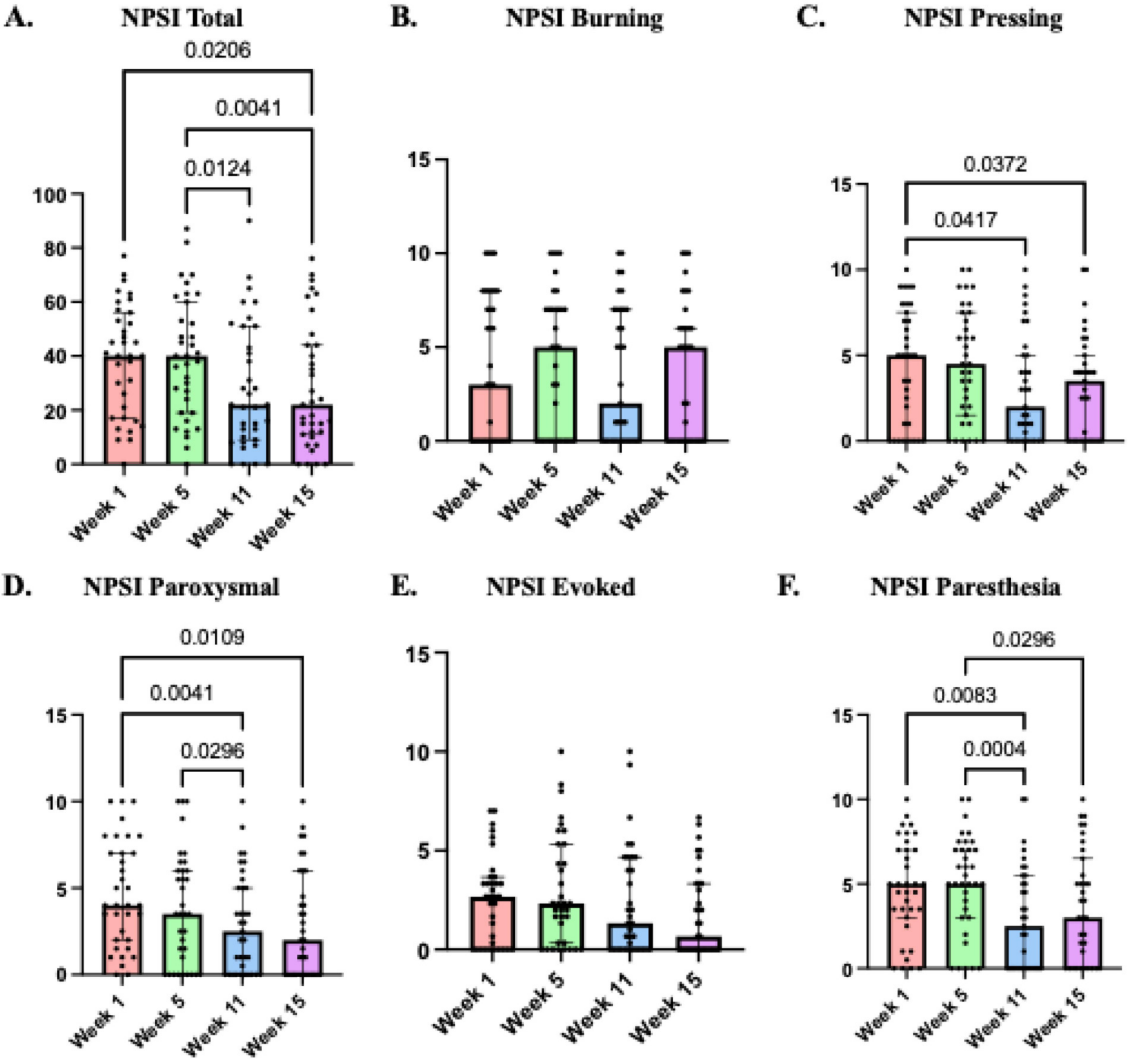
Neuropathic pain symptom inventory (NPSI) scores at weeks 1, 5, 11, and 15. **(A)** Summation of the total questionnaire (out of 100). **(B–F)** Clinically relevant dimensions of neuropathic pain syndromes were shown in their respective sub scores. *P*-values represented on the graph indicate Dunn's comparisons.

Consistent with participants' perspectives, quantitative pain outcomes improved. Non-parametric Friedman's ANOVA on NPSI total score showed a significant differences *χ^2^*(3 = 12.65, *p* = 0.006; Kendall's *W* = .12), with total NPSI scores decreasing from baseline (week 1) to week 15 and from week 5 to weeks 11 and 15. NPSI subscale scores, presented in panels B–F, also revealed significant reductions in clinically relevant domains, including pressing pain *χ^2^*(3 = 7.8, *p* = 0.05; Kendall's *W* = .07) with a decrease from week 1 to weeks 11 and 15, paroxysmal pain *χ^2^*(3 = 13.87, *p* = 0.003; Kendall's *W* = .13) with a decrease from week 1 to weeks 11 and 15 and from week 5 to week 11, and finally paresthesia/dysesthesia *χ*^2^(3 = 17.05, *p* = 0.001; Kendall's *W* = .16) with a decrease from week 1 to week 11 and from week 5 to weeks 11 and 15. Together with the qualitative findings these results indicate a meaningful reduction in neuropathic pain severity, particularly following the combined exercise and bodily illusion intervention (week 11), that remained at follow-up, as shown in Figure 4.

##### 3.3.1.3 Pain interference

(a) Qualitative

Participants reported less pain interference in their daily activities during and after the pain program in many domains such as their activity, sleep, and mood.

###### 3.3.1.3.1 Activity

Participants highlighted that the pain program directly enhanced their ability to manage daily life, promoting greater independence, confidence, and participation in meaningful activities. They reported that “*Pain didn't get in the way of completing other tasks”; “I last way longer throughout the day now”; “I would say it impacted it greatly a lot because now I could sit in the chair.”* Which opened the door to more active routines, “*I was able to fill my calendar with some more activity*” and *“Things I couldn't easily handle or couldn't handle it at all before, now I'm handling them on the lower level.”*

###### 3.3.1.3.2 Sleep

Several participants described experiencing improved sleep following exercise. One participant noted it was “*the best I would sleep at night. I would just rest at night*.*”* Others reported that “*when I come home, I can get a good rest*” and that the program “*gives you a better sleep, deeper”.* Another participant reported, “*I'm able to manage it better sleep”*, highlighting the perceived benefits of the program on their overall restfulness.

###### 3.3.1.3.3 Mood

Participants reported enhanced mood “*I start the day in another mood, I'm so happy. I'm telling you that I'm so proud of myself doing what I doing now. I'm stronger, I'm more motivated and I am another person right now.”,* and “*So my mood is a little better, not as irritable because I'm in pain or not as agitated, I'm not as bothered because I'm in pain all the time”.*

**Figure 5:**
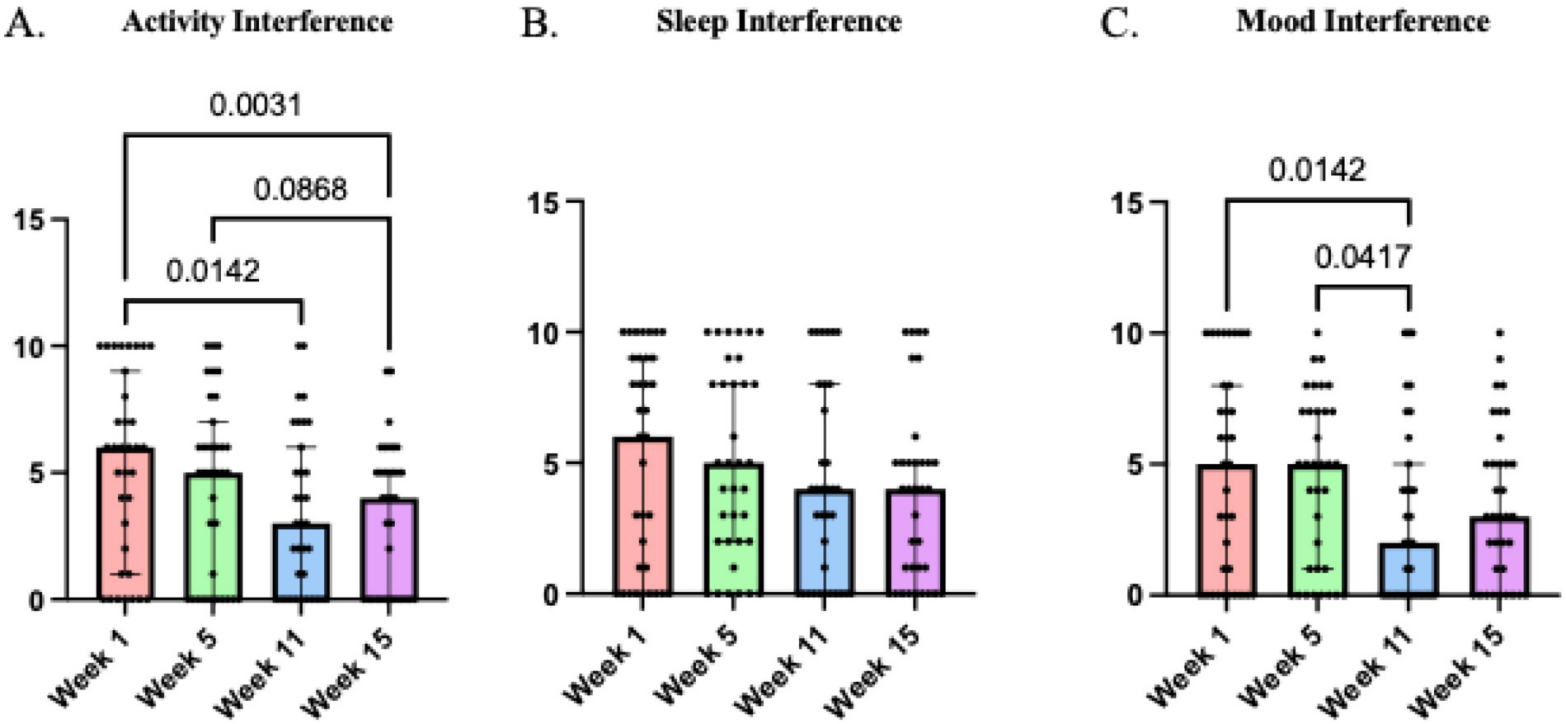
Interference in daily activities at weeks 1, 5, 11, and 15 of the multimodal pain program as indicated from the pain interference questionnaire: pain interfering with **(A)** activities, **(B)** sleep, and **(C)** mood. *P*-values represented on the graph indicate Dunn's comparisons.

(b) Quantitative:

Friedman's ANOVA showed a significant reduction in pain interference related to daily activities *χ^2^*(3 = 14.82, *p* = 0.002; Kendall's *W* = .14) (with a decrease from week 1 to weeks 11 and 15 and from week 5 to week 15), and mood *χ^2^*(3 = 9.925, *p* = 0.019; Kendall's *W* = .09) (with a decrease from week 1 to week 11 and from week 5–11). Sleep interference was not significantly decreased *χ^2^*(3 = 4.55, *p* = 0.208; Kendall's *W* = .04). Interferences were assessed via the pain interference questionnaire, see Figure 5.

##### 3.3.1.4 Better understanding of pain and pain management

(a) Qualitative

The goal of the pain program was to increase participants' ability to reduce their pain and its impact. Following the interventions, one participant said, “*Before I didn't know what to do to take away the pain or what to do to somewhat lessen the pain temporarily*,” but now feel like they are able to understand and manage their pain better, which was echoed through interviews with other participants “*It made me understand the pain more*” and “*Getting a broader understanding of my injury*. *Getting a broader understanding of just how the body process things, how the body heals, how the body try to..* *Just the body itself is an amazing tool*”. One participant noted, “*I liked the exercise, the pain relief, the knowledge that I was able to gain from the study. Most of all, it was just learning how to deal with pain and manage pain”.*

The results are likely an additive effect from the pain education, bodily illusion, and exercise, some participants highlighted the impact from the change in mindset *“Let's just do it. Let's just see where it leads”, “it gets you up. It gets you going so you don't always feel defeated and depleted”,* and *“Power of the mind works really well,”* and exercise *“I learned different ways to manage a pain as far as exercising,” “When next woke up, I feel a little tight. After I finish working out, I feel great. My body feel loose” and “now I'll just stretch myself cause it makes it feel good”.* Overall, participants felt that *“The study emotionally gave me more awareness and the understanding of why the pain is bothering me” and “helped me learn that I can manage my pain”.* Another benefit that came from this program was participants reporting that “*things I couldn't easily handle or couldn't handle it at all before, now I'm handling them on the lower level”* and that they were *“Really, really happy for what happened to me now”.* Although each participant has a unique, individualized pain, many participants expressed similar perspectives as this participant, *“So I mean, the situation comes with chronic pain, there's no way, there's no if, ands or buts about it, but the more you can manage it, the better your daily life can be.”*

(b) Qualitative/Quantitative

**Figure 6:**
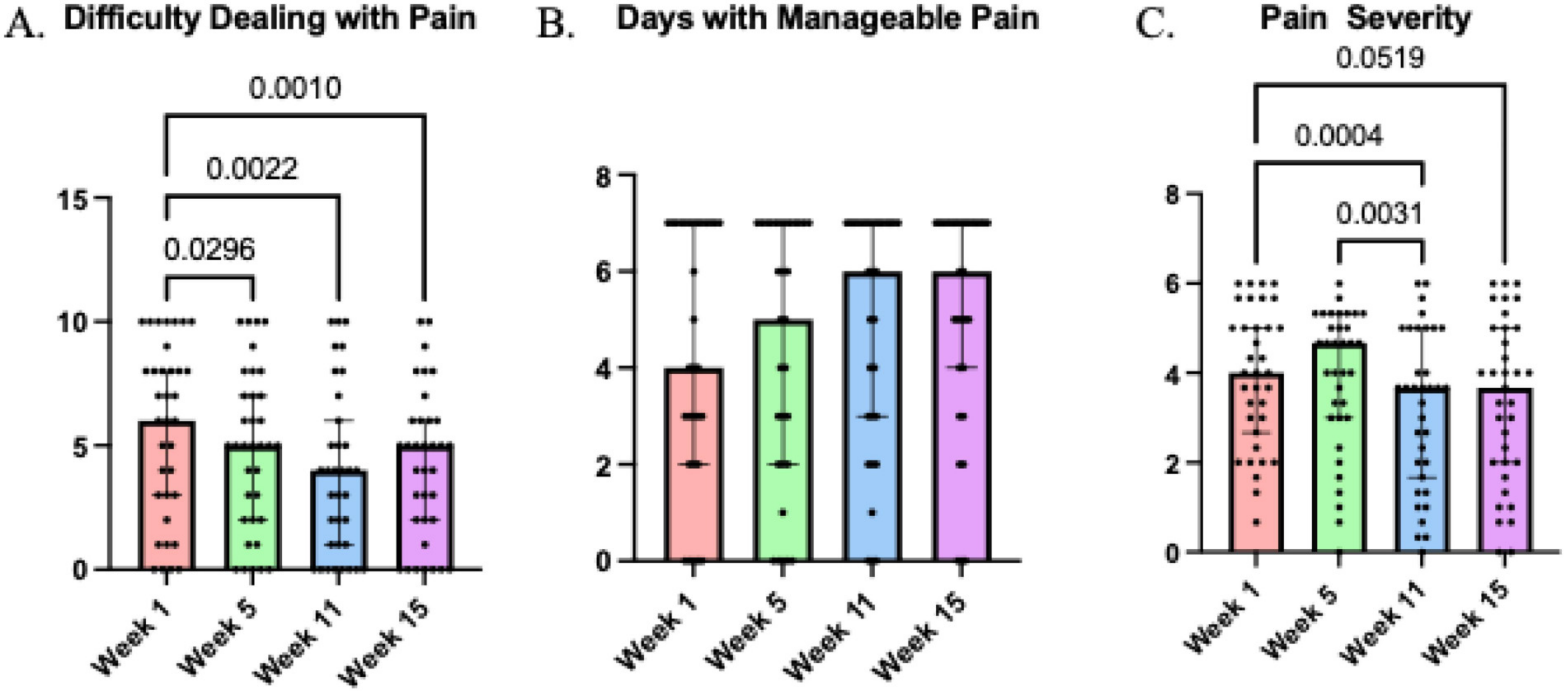
Participants responses to: **(A)** how difficult it is to deal with pain on a 0–10 scale; **(B)** how many days do you have manageable pain on a 0-7 scale; **(C)** overall pain severity, taken from the subscale of the MPI, throughout the 4 assessments in the pain program. *P*-values represented on the graph indicate Dunn's comparisons.

Participants reported a significant improvement in their ability to deal with pain *χ^2^*(3 = 16.14, *p* = 0.001; Kendall's *W* = .15) (Figure 6), as reflected in the question, “How difficult is it to deal with pain?”, with improvements maintained through week 15. Although the quantitative analysis of days with manageable pain did not reach significance, as shown in Figure 6B, there is a clear trend towards an increase in days with manageable pain throughout the pain program. Supporting this trend, participants gained “*new tools*” that “*helped me manage the pain even more”* and strategies that they could apply in their daily lives, “*I am more able to process the pain on daily basis.”*. For many, this program offered “*other options and ways that I could try to manage or control the pain,”* with one participant saying that they are “*implementing a lot of what I learned to my daily activities now to help reduce the pain that I have.”* Additionally, pain severity, a subscale of the Multidimensional Pain Inventory (MPI) (Figure 6C), showed a significant effect *χ^2^*(3 = 16.96, *p* < 0.0007; Kendall's *W* = .16), with a decrease from week 1 to weeks 11 and 15 and from week 5 to 11.

##### 3.3.1.5 Reduced dependence on medication or external aids

Participants expressed that this program allowed them to utilize other pain management options other than medication and external aids, with participants stating that, *“Before, I'd always be in a fetal position… now I do the workout instead”* and that despite having “*TENS unit, patches*” available, “*here I feel no pain*” during exercise sessions, suggesting that the exercise circuit provided relief. One participant also noted that they began “*taking a little less pain meds*” as their “*pain level was coming down*,” throughout the pain program.

#### 3.3.2 Barriers and limitations

Despite the positive outcomes reported by participants, there were barriers related to transportation, tiring interventions, lack of realism, negative impact from the walking illusion, other exercise preferences, triggering pain, already managing pain well, and temporary pain relief.

##### 3.3.2.1 Transportation challenges

Transportation challenges represented the most significant obstacle, with participants noting, “*The only thing is that I live an hour away. Just traveling there. Even though I like the workout part is I had to go twice a week and it was just a lot”* and “*the transportation was probably the hardest”*.

##### 3.3.2.2 Tiring/fatigue

Participants also noted that “*the whole study was a little long”*, which made it difficult to participate. For some participants, the exercise intervention was tiring, with some participants mentioning that they were “*Just draining myself out”* and “*very tired after the exercises”.*

##### 3.3.2.3 Lack of connection and realism

The walking illusion intervention faced some barriers that may have limited its impact. A primary challenge was participants' inability to maintain a connection with the illusion, with many experiencing cognitive disconnection from the virtual representation. As participants described, “*Don't feel like those are my legs*” and “*Hard to convince yourself that you're walking when you're just looking*,” highlighting the difficulty in achieving embodiment of the virtual limbs. Participants perceived the intervention as lacking utility or realism, expressing that it “*Didn't feel like it was too useful*” and “*Doesn't do much.*” and “*trying to elude myself into believing that these could be my legs actually moving like this. Those are my hopes, those are my dreams, that's what I'm striving for. But in the moment I'm grounded with the reality that this is what I'm dealing with”.* The intervention triggered emotional distress for some participants, evoking sadness and disappointment as one explained: “*I want to say it's sad, because it reminds you of what you should be doing.*”.

##### 3.3.2.4 Other exercise preferences

The intensity and duration of exercise sessions sometimes exceeded participants' physical capacity, “*Just draining myself out, not knowing my limitations*”. Specific exercise equipment proved problematic for certain participants, they reported “*Don't like the glider*” and discomfort with “*the rower*”. Some exercises triggered or exacerbated existing pain conditions in some of the participants. Participants experienced anxiety about potential injury, with one stating “*The risk for me was I would be sometimes scared of a certain movement or do something to hurt my back*,” while others reported direct pain increases: “*I was starting to feel lots of pain in my neck*”; “*some exercises in the study that were incorporated that aggravated my pain*” and “*end of the study my pain would be a little bit more flared up*” suggesting that for some individuals, the exercise intervention may have been counterproductive to the primary goal of pain management.

##### 3.3.2.5 Exacerbation or trigger of pain

Counterproductively, some participants experienced increased pain due to heightened focus on their paralyzed limbs, with one noting “*Almost made my pain worse because I was just sitting there and thinking about moving my own legs. But then that made it hurt more*” and “*I was thinking about my pain more.*” Additional physical discomfort also included motion sickness “*I got a little motion sick*”

##### 3.3.2.6 Already managing pain well

A subset of participants felt that were already implementing the pain management strategies, as one participant expressed, “*I've been managing the pain for so long already*,” while another noted, “*So though I learned ways to cope with my pain and how to manage it, I feel like I've been doing the same things on my own*.”

##### 3.3.2.7 Temporary pain relief

Additionally, the temporary pain relief posed a challenge for some participants, with one participant described this limitation by explaining that “*while I'm exercising, yes, it was good, but then throughout the whole day, the other day, it's just pain again,”*. These barriers indicate that while the program provided valuable benefits for many participants, future programs may need to incorporate more individualized pain management skills.

## 4 Discussion

The main objective of this mixed method study was to assess participants' perspectives on a non-pharmacological multimodal pain program in general and its effects on neuropathic pain. Participants reported multiple important benefits including positive effects on overall physical and mental health, engagement, and neuropathic pain and pain management. Importantly, these qualitative findings were congruent with statistically significant overall reductions in pain severity scores, improvement in psychosocial outcomes, and decreased pain interference with activities and mood scores.

In general, there has been an increased emphasis on multimodal therapeutic pain management (67–71). Multimodal programs focusing on chronic pain have utilized non-pharmacological interventions including pain education (50, 67, 72, 73), exercise (50, 72, 74–76), acupuncture (76), electrical stimulation (11, 14, 16, 20), bodily illusions (20, 26), virtual reality (77, 78), and cognitive behavioral therapies (50, 76, 79); individually or in combination. It appears that pain education is best utilized in combination with other treatment strategies (67, 73, 76). For example, programs incorporating only pain education found improvements in pain interference and depression (67) but when incorporated with other interventions, specific pain outcomes such as momentary pain (76) and pain interference (73), were significantly improved. Therefore, having an education resource and comprehensive knowledge of pain can impact participants perceptions of pain (50, 55. The initial phase of this study previously published (55), focused on pain education, likely significantly contributed to the overall positive results although only minimal effects on pain and psychosocial scores were observed directly after pain education. The perceived benefits of pain education included having a better understanding about pain, treatment options and self-management in general, learning from and interacting with peers, having a better understanding about pathophysiology of pain and being able to better communicate with healthcare providers, family, and friends. In addition, the pain education had a significant positive impact on participants difficulty in dealing with pain scores, supporting the idea that pain education can facilitate the adjustment to living with chronic pain after SCI and possibly reduce some of psychological distress associated with experiencing persistent neuropathic pain (80). Consistent with this idea, affective distress is known to be significantly linked to chronic pain that has a high impact on life after SCI (81) and therefore a reduction in pain impact is likely also reducing the emotional impact. Previous research has shown that a combination of perceived greater pain impact and limitations, difficult nature of pain, poor communication from provider, lower resilience, greater medication use, and younger age predicts greater difficulty in dealing with pain after SCI (82). This highlights both the complexity in not only underlying mechanisms of pain after SCI but also the intricate association with multiple psychosocial domains.

Many people with SCI-related neuropathic pain utilize multiple self-remedies to manage their pain effectively (83). In the present study, participants reported that they frequently managed their pain using distraction and shifting focus to something else. Distraction as a tool for managing pain has been reported in studies involving both SCI (82) and other chronic pain populations (84). Indeed, our results show that during both exercise and walking illusion, some participants noted that they gradually developed a skill where they mentally could distract themselves from their pain and redirect their focus to the present activity. These findings are consistent with other studies reporting significant reductions in both pain interference and pain-related anxiety following mindfulness (85, 86) and cognitive engagement (84) interventions. Additionally, coping skills have been shown to predict daily management of pain following SCI (83, 87, 88). This concurs with the positive reports of managing pain in the present study and the significant reductions in difficulty dealing with pain. Although number of days with manageable pain were not significantly increased post exercise and walking illusion, there was a trend towards having more days with manageable pain. The lack of significant improvement was probably related to the fact that a large proportion of our participants, despite experiencing moderate to severe neuropathic pain, felt that they had manageable pain every day already at the baseline assessment. Because of the refractory nature of neuropathic pain, making pain manageable is a more realistic goal than complete amelioration. However, the meaning of manageable pain in people with SCI varies widely among individuals. A recent study in SCI (49) found that manageable pain was generally characterized by moderate pain intensity, predictability, low interference with daily activities, and pain not requiring medication (87, 88).

Congruent with this, we found a significant improvement with pain interference across both qualitative reports and quantitative scores. Several participants reported that they were able to fill their calendar with activities and complete tasks without being limited by their pain, which was also supported by the significant decreases in pain interference scores. A reduction in pain interference with daily activity has a profound impact on QOL following SCI (89) and our results highlight the importance of reducing pain interference to improve daily functioning. Additionally, some participants reported having better sleep, which was supported by a slight non-significant average improvement in pain interference with sleep scores. The significant decrease in pain interference with mood was paralleled by some participants reported starting the day in a better mood than before.

Other benefits reported by some participants included that they felt as though they had newfound control of their life and expanded mindset regarding what they could do. Others felt that this allowed them to reduce the limitations they put on themselves. Along with the confidence to accomplish more, participants reported their own self-image improved. This has been shown to impact the success of pain self-management, due to participants motivation and readiness to change (90). Increased motivation and continuing to exercise after the end of the study was reported by most participants at the follow-up interview, with some emphasizing that the positive experience of participating in the study changed the way they viewed their future. These positive views were also supported by the retention of significant improvements at the follow-up assessments for NPSI total and sub scores, pain interference with activities, and difficulty dealing with pain.

It is widely accepted that social interactions and communication may influence individuals perceptions of pain and responsiveness to therapeutic interventions (91). Indeed, some participants noted the positive impact of the community and interpersonal connections throughout the program. In the SCI community, research supports that social support directly impacts QOL and can influence the negative effects of pain on QOL (92). For example, prior to the study, some participants reported feeling isolated due to their injury and emphasized the significant and positive impact of connecting with others with SCI during the study and valued the opportunity to exchange knowledge and resources with each other.

Finally, although our study combined exercise and walking illusion, we believe that exercise had some specific beneficial effects. Specific to the exercise intervention, some participants noted that their increase in strength and stamina had an overall health impact. Beyond the noticeable change in upper-extremity strength, participants noticed an improvement in core strength, which allowed them to maintain better posture and have better balance throughout the day. Other participants reported an improvement in their cardiovascular health, with two participants improving blood pressure and cholesterol levels. Participants also felt as though they benefited from the personalized exercise, with many reporting that they were able to push themselves and improve weight in subsequent weeks because of the adaptations and instructions provided at the beginning of the intervention. Previous studies have shown that exercise has a wide range of beneficial effects for people with SCI (93). However, the optimal level of intensity, duration, and type of exercise specifically for pain management still being debated (75, 94). Upper limb circuit training has been shown to have significant improvements in strength, endurance, cardiometabolic markers, and pain outcomes (58, 95, 96). Several studies in SCI highlight the benefits of exercise on cardiometabolic health (97–100); which was consistent with lower blood pressure and cholesterol levels mentioned by participants in the present study. However, some studies also report conflicting results regarding the effects of exercise on physiological and pain outcomes. For example, while some studies report improvements on QOL (101, 102), and pain outcomes in chronic pain populations (28–32), others show no clinically significant changes in QOL, physiological state (74), and pain outcomes (74). However, the results from the present study are consistent with literature supporting evidence that exercise-based interventions can lead to measurable improvements in pain. As mentioned earlier, our program included both exercise and walking illusion. This latter has previously been shown to reduce neuropathic pain intensity in SCI individuals, either alone (15) or in combination with tDCS (16, 20). However, these studies only focused on quantitative pain outcomes, mainly a reduction in pain intensity, and did not consider participants' perspectives on these types of interventions. In the present study we aimed to significantly expand on these prior studies and included extensive qualitative interviews exploring participants' perspectives on a wide range of aspects of the study. We show that some participants experienced the walking illusion as a convincing simulation of movement that enhanced bodily ownership and agency. The intervention also elicited relaxation and positive mood, with some participants continuing the imagery practice outside the sessions. However, some negative perspectives were also provided by participants. For instance, some participants reported that the illusion felt unrealistic or, in some cases, triggered negative emotions by highlighting their lost abilities. These reports open the way for improvement in future trials using similar non-invasive interventions.

In conclusion, the outcomes from the present study provide support for non-pharmacological approaches that can be combined with pharmacological treatments for pain when needed. With the wide range of positive benefits reported in the qualitative interviews and the significant improvements in pain outcomes, pain interference in daily activities, this multimodal pain program successfully helped some individuals manage their neuropathic pain. However, some barriers emerged. For instance, transportation was a frequently mentioned significant difficulty for the participation in the study. Therefore, future studies need to take into consideration at-home options for non-pharmacological treatments, in this way access to these types of programs can be beneficial also for those individuals unable to attend multiple study sessions in experimental settings. Additionally, some suggested the use of a more realistic walking illusion using virtual reality (VR). The use of VR has recently been utilized as a non-pharmacological intervention for neuropathic pain and chronic pain populations (77, 78, 103, 104) via the remapping of body representation (104) and distraction from pain (103). It would be beneficial to develop VR interventions that can be home-based and consider participants' preferences for enhanced realism. Future studies should therefore focus on individually targeted and integrated combinations of interventions to improve overall outcomes.

Although this pain program showed meaningful benefits, the study also presents some limitations that must be acknowledged. This study was adequately powered for qualitative analysis, it was not powered for quantitative analyses, and we did not include a comparison intervention. This means that while the sample size was sufficient to generate reliable themes from participants' interviews, the quantitative findings may not be robust enough to be generalized to a broader SCI population, and the missing comparison intervention may introduce some bias in the results. Our sample also included a larger percentage of male participants, and although this is representative of the SCI population in the US (105), this may present a limitation for the generalizability of the results to women with SCI. Additionally, we did not include individuals with mild neuropathic pain or those with severe depression, and this limits our understanding of whether these benefits also apply to individuals with mild pain or severe depression. Finally, we believe that future studies including multidimensional pain programs similar to ours may have a benefit on psychosocial aspects and although our participants reported changes in their mood in the qualitative interviews, we did not include a more comprehensive mood or psychosocial assessment that might have further supported these reports.

In conclusion, the combination of both qualitative data with pain outcome measures strongly supports the usefulness of the present non-pharmacological multimodal pain program for many who experience moderate to severe neuropathic pain long after injury.

## Data Availability

The original contributions presented in the study are included in the article/Supplementary Material, further inquiries can be directed to the corresponding author.

## References

[1] Finnerup NB Norrbrink C Trok K Piehl F Johannesen IL Sørensen JC Phenotypes and predictors of pain following traumatic spinal cord injury: a prospective study. J Pain. (2014) 15(1):40–8. 10.1016/j.jpain.2013.09.008 24268112

[2] Andresen SR Biering-Sørensen F Hagen EM Nielsen JF Bach FW Finnerup NB. Pain, spasticity and quality of life in individuals with traumatic spinal cord injury in Denmark. Spinal Cord. (2016) 54(11):973–9. 10.1038/sc.2016.46 27067654

[3] Loh E Mirkowski M Agudelo AR Allison DJ Benton B Bryce TN The CanPain SCI clinical practice guidelines for rehabilitation management of neuropathic pain after spinal cord injury: 2021 update. Spinal Cord. (2022) 60(6):548–66. 10.1038/s41393-021-00744-z 35124700 PMC9209331

[4] Cardenas DD Nieshoff EC Suda K Goto SI Sanin L Kaneko T A randomized trial of pregabalin in patients with neuropathic pain due to spinal cord injury. Neurology. (2013) 80(6):533–9. 10.1212/WNL.0b013e318281546b 23345639 PMC3589291

[5] Onakpoya IJ Thomas ET Lee JJ Goldacre B Heneghan CJ. Benefits and harms of pregabalin in the management of neuropathic pain: a rapid review and meta-analysis of randomised clinical trials. BMJ Open. (2019) 9(1):e023600. 10.1136/bmjopen-2018-023600 30670513 PMC6347863

[6] Polat CS Konak HE Akıncı MG Onat SS Altas EU. Misuse of gabapentinoids (pregabalin and gabapentin) in patients with neuropathic pain related to spinal cord injury. J Spinal Cord Med. (2023) 46(5):859–64. 10.1080/10790268.2021.2024709 35108174 PMC10446779

[7] Widerstrom-Noga E Anderson KD Perez S Martinez-Arizala A Calle-Coule L Fleming L. Barriers and facilitators to optimal neuropathic pain management: sCI consumer, significant other, and health care provider perspectives. Pain Med Malden Mass. (2020) 21(11):2913–24. 10.1093/pm/pnaa058 32219441

[8] Kesikburun S. Non-invasive brain stimulation in rehabilitation. Turk J Phys Med Rehabil. (2022) 68(1):1–8. 10.5606/tftrd.2022.10608 35949977 PMC9305642

[9] Zhao Q Zhao L Fan P Zhu Y Zhu R Cheng L Non-invasive TMS attenuates neuropathic pain after spinal cord injury associated with enhancing brain functional connectivity and HPA axis activity. Heliyon. (2024) 10(16):e36061. 10.1016/j.heliyon.2024.e36061 39253232 PMC11382048

[10] García-Alén L Ros-Alsina A Sistach-Bosch L Wright M Kumru H. Noninvasive electromagnetic neuromodulation of the central and peripheral nervous system for upper-limb motor strength and functionality in individuals with cervical spinal cord injury: a systematic review and meta-analysis. Sensors. (2024) 24(14):4695. 10.3390/s24144695 39066092 PMC11280769

[11] Gibson W Wand BM O’Connell NE. Transcutaneous electrical nerve stimulation (TENS) for neuropathic pain in adults. Cochrane Database Syst Rev. (2017). 10.1002/14651858.cd011976.pub2 PMC6426434 28905362

[12] Nnoaham KE Kumbang J. Transcutaneous electrical nerve stimulation (TENS) for chronic pain. Cochrane Database Syst Rev. (2008) (3):CD003222. doi: 10.1002/14651858.CD003222.pub2 10.1002/14651858.CD003222.pub2 18646088

[13] Fregni F Gimenes R Valle AC Ferreira MJL Rocha RR Natalle L A randomized, sham-controlled, proof of principle study of transcranial direct current stimulation for the treatment of pain in fibromyalgia. Arthritis Rheum. (2006) 54(12):3988–98. 10.1002/art.22195 17133529

[14] Ngernyam N Jensen MP Arayawichanon P Auvichayapat N Tiamkao S Janjarasjitt S The effects of transcranial direct current stimulation in patients with neuropathic pain from spinal cord injury. Clin Neurophysiol Off J Int Fed Clin Neurophysiol. (2015) 126(2):382–90. 10.1016/j.clinph.2014.05.034 25027640

[15] Moseley LG. Using visual illusion to reduce at-level neuropathic pain in paraplegia. Pain. (2007) 130(3):294–8. 10.1016/j.pain.2007.01.007 17335974

[16] Soler MD Kumru H Pelayo R Vidal J Tormos JM Fregni F Effectiveness of transcranial direct current stimulation and visual illusion on neuropathic pain in spinal cord injury. Brain J Neurol. (2010) 133(9):2565–77. 10.1093/brain/awq184 PMC2929331 20685806

[17] Widerström-Noga E. Neuropathic pain and spinal cord injury: management, phenotypes, and biomarkers. Drugs. (2023) 83(11):1001–25. 10.1007/s40265-023-01903-7 37326804

[18] Geneen LJ Martin DJ Adams N Clarke C Dunbar M Jones D Effects of education to facilitate knowledge about chronic pain for adults: a systematic review with meta-analysis. Syst Rev. (2015) 4:132. 10.1186/s13643-015-0120-5 26428467 PMC4591560

[19] Widerström-Noga E Anderson KD Robayo LE Perez S Martinez-Arizala A Calle-Coule L Development of a pain education resource for people with spinal cord injury. Front Public Health. (2023) 11:1197944. 10.3389/fpubh.2023.1197944 37554730 PMC10406314

[20] Soler D Moriña D Kumru H Vidal J Navarro X. Transcranial direct current stimulation and visual illusion effect according to sensory phenotypes in patients with spinal cord injury and neuropathic pain. J Pain. (2021) 22(1):86–96. 10.1016/j.jpain.2020.06.004 32629032

[21] Eick J Richardson EJ. Cortical activation during visual illusory walking in persons with spinal cord injury: a pilot study. Arch Phys Med Rehabil. (2015) 96(4):750–3. 10.1016/j.apmr.2014.10.020 25461820 PMC4380793

[22] Vastano R Costantini M Widerstrom-Noga E. Maladaptive reorganization following SCI: the role of body representation and multisensory integration. Prog Neurobiol. (2022) 208:102179. 10.1016/j.pneurobio.2021.102179 34600947

[23] Henderson LA Gustin SM Macey PM Wrigley PJ Siddall PJ. Functional reorganization of the brain in humans following spinal cord injury: evidence for underlying changes in cortical anatomy. J Neurosci. (2011) 31(7):2630–7. 10.1523/JNEUROSCI.2717-10.2011 21325531 PMC6623700

[24] Frank SI Mylavarapu RV Widerstrom-Noga E Vastano R. Early body representation EEG signals in cervical vs. Thoracic spinal cord injuries with neuropathic pain. Brain Res. (2025) 1858:149658. 10.1016/j.brainres.2025.149658 40286834

[25] Vastano R Widerstrom-Noga E. Event-related potentials during mental rotation of body-related stimuli in spinal cord injury population. Neuropsychologia. (2023) 179:108447. 10.1016/j.neuropsychologia.2022.108447 36521630

[26] Moro V Scandola M Aglioti SM. What the study of spinal cord injured patients can tell us about the significance of the body in cognition. Psychon Bull Rev. (2022) 29(6):2052–69. 10.3758/s13423-022-02129-6 35697914 PMC9722882

[27] Ionta S Villiger M Jutzeler CR Freund P Curt A Gassert R. Spinal cord injury affects the interplay between visual and sensorimotor representations of the body. Sci Rep. (2016) 6:20144. 10.1038/srep20144 PMC4740737 26842303

[28] Andersen Hammond E Pitz M Shay B. Neuropathic pain in taxane-induced peripheral neuropathy: evidence for exercise in treatment. Neurorehabil Neural Repair. (2019) 33(10):792–9. 10.1177/1545968319860486 31342880

[29] Dhawan S Andrews R Kumar L Wadhwa S Shukla G. A randomized controlled trial to assess the effectiveness of muscle strengthening and balancing exercises on chemotherapy-induced peripheral neuropathic pain and quality of life among cancer patients. Cancer Nurs. (2020) 43(4):269–80. 10.1097/NCC.0000000000000693 30888982

[30] Demaneuf T Aitken Z Karahalios A Leong TI Livera D Jelinek AM Effectiveness of exercise interventions for pain reduction in people with multiple sclerosis: a systematic review and meta-analysis of randomized controlled trials. Arch Phys Med Rehabil. (2019) 100(1):128–39. 10.1016/j.apmr.2018.08.178 30240593

[31] Ambrose KR Golightly YM. Physical exercise as non-pharmacological treatment of chronic pain: why and when. Best Pract Res Clin Rheumatol. (2015) 29(1):120–30. 10.1016/j.berh.2015.04.022 26267006 PMC4534717

[32] Wylde V Dennis J Beswick AD Bruce J Eccleston C Howells N Systematic review of management of chronic pain after surgery. Br J Surg. (2017) 104(10):1293–306. 10.1002/bjs.10601 28681962 PMC5599964

[33] Lavin KM Coen PM Baptista LC Bell MB Drummer D Harper SA State of knowledge on molecular adaptations to exercise in humans: historical perspectives and future directions. Compr Physiol. (2022) 12(2):3193. 10.1002/j.2040-4603.2022.tb00211.x 35578962 PMC9186317

[34] Cooper MA Kluding PM Wright DE. Emerging relationships between exercise, sensory nerves, and neuropathic pain. Front Neurosci. (2016) 10:372. 10.3389/fnins.2016.00372 27601974 PMC4993768

[35] Lima LV Abner TSS Sluka KA. Does exercise increase or decrease pain? Central mechanisms underlying these two phenomena. J Physiol. (2017) 595(13):4141–50. 10.1113/JP273355 28369946 PMC5491894

[36] Sandrow-Feinberg HR Houlé JD. Exercise after spinal cord injury as an agent for neuroprotection, regeneration and rehabilitation. Brain Res. (2015) 1619:12–21. 10.1016/j.brainres.2015.03.052 25866284 PMC4540698

[37] Kuphal KE Fibuch EE Taylor BK. Extended swimming exercise reduces inflammatory and peripheral neuropathic pain in rodents. J Pain. (2007) 8(12):989–97. 10.1016/j.jpain.2007.08.001 17890162

[38] Kodesh E Weissman-Fogel I. Exercise-induced hypoalgesia—interval versus continuous mode. Appl Physiol Nutr Metab Physiol Appl Nutr Metab. (2014) 39(7):829–34. 10.1139/apnm-2013-0481 24773287

[39] Rice D Nijs J Kosek E Wideman T Hasenbring MI Koltyn K Exercise-Induced hypoalgesia in pain-free and chronic pain populations: state of the art and future directions. J Pain. (2019) 20(11):1249–66. 10.1016/j.jpain.2019.03.005 30904519

[40] Kami K Tajima F Senba E. Exercise-induced hypoalgesia: potential mechanisms in animal models of neuropathic pain. Anat Sci Int. (2017) 92(1):79–90. 10.1007/s12565-016-0360-z 27484434

[41] Henriksen M Klokker L Graven-Nielsen T Bartholdy C Schjødt Jørgensen T Bandak E Association of exercise therapy and reduction of pain sensitivity in patients with knee osteoarthritis: a randomized controlled trial. Arthritis Care Res. (2014) 66(12):1836–43. 10.1002/acr.22375 24905427

[42] Tweedy SM Beckman EM Geraghty TJ Theisen D Perret C Harvey LA Exercise and sports science Australia (ESSA) position statement on exercise and spinal cord injury. J Sci Med Sport. (2017) 20(2):108–15. 10.1016/j.jsams.2016.02.001 27185457

[43] Nash MS Groah SL Gater DR Dyson-Hudson TA Lieberman JA Myers J Identification and management of cardiometabolic risk after spinal cord injury: clinical practice guideline for health care providers. Top Spinal Cord Inj Rehabil. (2018) 24(4):379–423. 10.1310/sci2404-379 30459501 PMC6241225

[44] Nash MS Jacobs PL Mendez AJ Goldberg RB. Circuit resistance training improves the atherogenic lipid profiles of persons with chronic paraplegia. J Spinal Cord Med. (2001) 24(1):2–9. 10.1080/10790268.2001.11753548 11587430

[45] Piira A Lannem AM Gjesdal K Knutsen R Jørgensen L Glott T Quality of life and psychological outcomes of body-weight supported locomotor training in spinal cord injured persons with long-standing incomplete lesions. Spinal Cord. (2020) 58(5):560–9. 10.1038/s41393-019-0401-2 31848443

[46] Hicks AL Martin KA Ditor DS Latimer AE Craven C Bugaresti J Long-term exercise training in persons with spinal cord injury: effects on strength, arm ergometry performance and psychological well-being. Spinal Cord. (2003) 41(1):34–43. 10.1038/sj.sc.3101389 12494319

[47] Allen JM Berg Miller ME Pence BD Whitlock K Nehra V Gaskins HR Voluntary and forced exercise differentially alters the gut microbiome in C57BL/6J mice. J Appl Physiol Bethesda Md 1985. (2015) 118(8):1059–66. 10.1152/japplphysiol.01077.2014 25678701

[48] Norman C Bender JL Macdonald J Dunn M Dunne S Siu B Questions that individuals with spinal cord injury have regarding their chronic pain: a qualitative study. Disabil Rehabil. (2010) 32(2):114–24. 10.3109/09638280903033248 19817663

[49] Wong ML Anderson KD Roach KE Robayo L Cherup NP Vastano R The meaning of manageable neuropathic pain after SCI. Front Pain Res Lausanne Switz. (2025) 6:1540395. 10.3389/fpain.2025.1540395 PMC12187659 40568186

[50] Norrbrink Budh C Kowalski J Lundeberg T. A comprehensive pain management programme comprising educational, cognitive and behavioural interventions for neuropathic pain following spinal cord injury. J Rehabil Med. 2006 38(3):172–80. 10.1080/16501970500476258 16702084

[51] Arnau RC Meagher MW Norris MP Bramson R. Psychometric evaluation of the beck depression inventory-II with primary care medical patients. Health Psychol Off J Div Health Psychol Am Psychol Assoc. (2001) 20(2):112–9. 10.1037//0278-6133.20.2.112 11315728

[52] Scarpina F Migliorati D Marzullo P Mauro A Scacchi M Costantini M. Altered multisensory temporal integration in obesity. Sci Rep. (2016) 6(1):28382. 10.1038/srep28382 27324727 PMC4914987

[53] Babor TF Higgins-Biddle JC Saunders JB Monteiro MG. The Alcohol Use Disorder Identification Test (AUDIT): Guidelines for Use in Primary Care. 2nd ed. Geneva: World Health Organization (2001).

[54] Jacobs PL. Effects of resistance and endurance training in persons with paraplegia. Med Sci Sports Exerc. (2009) 41(5):992–7. 10.1249/MSS.0b013e318191757f 19346989

[55] Cherup NP Anderson KD Wong ML Fernandez GE Robayo LE Roach K Impact of a pain education program for people with spinal cord injury who experience neuropathic pain. Front Pain Res. (2025) 6. 10.3389/fpain.2025.1569446/full PMC12148921 40496137

[56] Fernandez GE Anderson KD Vastano R Frank SI Robayo LE Cherup NP Perspectives of people with spinal cord injury on a pain education resource. Front Public Health. (2024) 12:1385831. 10.3389/fpubh.2024.1385831 38962773 PMC11220275

[57] Jacobs PL Nash MS Rusinowski JW. Circuit training provides cardiorespiratory and strength benefits in persons with paraplegia. Med Sci Sports Exerc. (2001) 33(5):711–7. 10.1097/00005768-200105000-00005 11323537

[58] Nash MS van de Ven I van Elk N Johnson BM. Effects of circuit resistance training on fitness attributes and upper-extremity pain in middle-aged men with paraplegia. Arch Phys Med Rehabil. (2007) 88(1):70–5. 10.1016/j.apmr.2006.10.003 17207678

[59] Williams N. The borg rating of perceived exertion (RPE) scale. Occup Med. (2017) 67(5):404–5. 10.1093/occmed/kqx063

[60] Widerström-Noga E Biering-Sørensen F Bryce TN Cardenas DD Finnerup NB Jensen MP The international spinal cord injury pain basic data set (version 2.0). Spinal Cord. (2014) 52(4):282–6. 10.1038/sc.2014.4 24469147

[61] Bouhassira D Attal N Fermanian J Alchaar H Gautron M Masquelier E Development and validation of the neuropathic pain symptom inventory. Pain. (2004) 108:248–57. 10.1016/j.pain.2003.12.024 15030944

[62] Wong ML Fleming L Robayo LE Widerström-Noga E. Utility of the Neuropathic Pain Symptom Inventory in people with spinal cord injury. Spinal Cord. (2020) 58(1):35–42. 10.1038/s41393-019-0338-5 31431674

[63] Widerström-Noga EG Cruz-Almeida Y Martinez-Arizala A Turk DC. Internal consistency, stability, and validity of the spinal cord injury version of the multidimensional pain inventory. Arch Phys Med Rehabil*.* (2006) 87(4):516–23. 10.1016/j.apmr.2005.12.036 16571391

[64] Monrouxe LV Rees CE. When I say … quantification in qualitative research. Med Educ. (2020) 54(3):186–7. 10.1111/medu.14010 31795010

[65] Marshall B Cardon P Poddar A Fontenot R. Does sample size matter in qualitative research?: a review of qualitative interviews in is research. J Comput Inf Syst. (2013) 54(1):11–22. 10.1080/08874417.2013.11645667

[66] Charmaz K. Grounded theory. In: Ponzetti JJ Jr, editor. The Blackwell Encyclopedia of Sociology. Malden, MA: John Wiley & Sons, Ltd (2007). 10.1002/9781405165518.wbeosg070

[67] Bombardier CH Fann JR Ehde DM Reyes MR Burns SP Barber JK Collaborative care versus usual care to improve quality of life, pain, depression, and physical activity in outpatients with spinal cord injury: the SCI-CARE randomized controlled clinical trial. J Neurotrauma. (2023) 40(23–24):2667–79. 10.1089/neu.2023.0200 37597201 PMC11075937

[68] Williams TL Nilsson Wikmar L Joseph C. Principles for chronic pain management in the adult traumatic spinal cord injury population at the primary healthcare level, in a developing context: a Delphi study. Glob Adv Integr Med Health. (2025) 14:27536130251349456. 10.1177/27536130251349456 40630420 PMC12235114

[69] Proulx K Lamontagne ME Quirion R Deaudelin I Mercier C Perreault K. A six-participant pilot single-subject study of an individualized pain management program for people with spinal cord injury. Spinal Cord Ser Cases. (2023) 9(1):2. 10.1038/s41394-022-00557-z 36646690 PMC9842717

[70] Williams TL Joseph C Nilsson-Wikmar L Phillips J. Guidelines for chronic pain in adult spinal cord injury population: scoping review. South Afr J Physiother. (2024) 80(1):1931. 10.4102/sajp.v80i1.1931 PMC11151359 38841594

[71] Kamper SJ Apeldoorn AT Chiarotto A Smeets RJEM Ostelo RW Guzman J Multidisciplinary biopsychosocial rehabilitation for chronic low back pain. Cochrane Library. (2014). 10.1002/14651858.CD000963.pub3/full PMC10945502 25180773

[72] Scascighini L Toma V Dober-Spielmann S Sprott H. Multidisciplinary treatment for chronic pain: a systematic review of interventions and outcomes. Rheumatology. (2008) 47(5):670–8. 10.1093/rheumatology/ken021 18375406

[73] Katz L Patterson L Zacharias R. Evaluation of an interdisciplinary chronic pain program and predictors of readiness for change. Can J Pain. (2019) 3(1):70–8. 10.1080/24740527.2019.1582296 35005395 PMC8730559

[74] Akkurt H Karapolat HU Kirazli Y Kose T. The effects of upper extremity aerobic exercise in patients with spinal cord injury: a randomized controlled study. Eur J Phys Rehabil Med. (2017) 53(2):219–27. 10.23736/S1973-9087.16.03804-1 27824234

[75] Pelletier C. Exercise prescription for persons with spinal cord injury: a review of physiological considerations and evidence-based guidelines. Appl Physiol Nutr Metab Physiol Appl Nutr Metab. (2023) 48(12):882–95. 10.1139/apnm-2023-0227 37816259

[76] Skelly AC Chou R Dettori JR Turner JA Friedly JL Rundell SD Noninvasive Nonpharmacological Treatment for Chronic Pain: A Systematic Review. Rockville, MD: Agency for Healthcare Research and Quality (AHRQ). (2018). 30179389

[77] Pozeg P Palluel E Ronchi R Solcà M Al-Khodairy AW Jordan X Virtual reality improves embodiment and neuropathic pain caused by spinal cord injury. Neurology. (2017) 89(18):1894–903. 10.1212/WNL.0000000000004585 28986411 PMC5664293

[78] Selvaraj JS Prakash H Augustine TA Kumar SSK Velkumar S Amalraj JA. Immersive virtual reality for neuropathic pain management in spinal cord injury: a randomized controlled trial. J Spinal Cord Med. (2025):1–13. 10.1080/10790268.2025.2514321 PMC13123074 40638262

[79] Kent P Haines T O'Sullivan P Smith A Campbell A Schutze R Cognitive functional therapy with or without movement sensor biofeedback versus usual care for chronic, disabling low back pain (RESTORE): a randomised, controlled, three-arm, parallel group, phase 3, clinical trial. Lancet*.* (2023) 401(10391):1866–77. 10.1016/S0140-6736(23)00441-5 37146623

[80] Hanley MA Raichle K Jensen M Cardenas DD. Pain catastrophizing and beliefs predict changes in pain interference and psychological functioning in persons with spinal cord injury. J Pain. (2008) 9(9):863–71. 10.1016/j.jpain.2008.04.008 18550442 PMC2600516

[81] Widerström-Noga E Anderson KD Perez S Martinez-Arizala A Cambridge JM. Subgroup perspectives on chronic pain and its management after spinal cord injury. J Pain. (2018) 19(12):1480–90. 10.1016/j.jpain.2018.07.003 30056113

[82] Widerström-Noga E Anderson KD Perez S Hunter JP Martinez-Arizala A Adcock JP Living with chronic pain after spinal cord injury: a mixed-methods study. Arch Phys Med Rehabil. (2017) 98(5):856–65. 10.1016/j.apmr.2016.10.018 27894730

[83] Molton IR Stoelb BL Jensen MP Ehde DM Raichle KA Cardenas DD. Psychosocial factors and adjustment to chronic pain in spinal cord injury: replication and cross-validation. J Rehabil Res Dev. (2009) 46(1):31–42. 10.1682/JRRD.2008.03.0044 19533518 PMC2743728

[84] Rischer KM González-Roldán AM Montoya P Gigl S Anton F van der Meulen M. Distraction from pain: the role of selective attention and pain catastrophizing. Eur J Pain Lond Engl. (2020) 24(10):1880–91. 10.1002/ejp.1634 PMC7689692 32677265

[85] Hearn JH Finlay KA. Internet-delivered mindfulness for people with depression and chronic pain following spinal cord injury: a randomized, controlled feasibility trial. Spinal Cord. (2018) 56(8):750–61. 10.1038/s41393-018-0090-2 29581519

[86] Elomaa MM de Williams AC Kalso EA. Attention management as a treatment for chronic pain. Eur J Pain. (2009) 13(10):1062–7. 10.1016/j.ejpain.2008.12.002 19144553

[87] Heutink M Post M Overdulve C Pfennings L van de Vis W Vrijens N Which pain coping strategies and cognitions are associated with outcomes of a cognitive behavioral intervention for neuropathic pain after spinal cord injury? Top Spinal Cord Inj Rehabil. (2013) 19(4):330–40. 10.1310/sci1904-330 24244098 PMC3816727

[88] Widerström-Noga EG Cuervo F Broton E Duncan JG Yezierski RP. Perceived difficulty in dealing with consequences of spinal cord injury. Arch Phys Med Rehabil. (1999) 80(5):580–6. 10.1016/S0003-9993(99)90203-4 10326925

[89] Molton IR Jensen MP Nielson W Cardenas D Ehde DM. A preliminary evaluation of the motivational model of pain self-management in persons with spinal cord injury related pain. J Pain Off J Am Pain Soc. (2008) 9(7):606–12. 10.1016/j.jpain.2008.01.338 PMC2493418 18359668

[90] Molton IR Jensen MP Nielson W Cardenas D Ehde DM. A preliminary evaluation of the motivational model of pain self-management in persons with spinal cord injury related pain. J Pain Off J Am Pain Soc. (2008) 9(7):606–12. 10.1016/j.jpain.2008.01.338 PMC2493418 18359668

[91] Karayannis NV Baumann I Sturgeon JA Melloh M Mackey SC. The impact of social isolation on pain interference: a longitudinal study. Ann Behav Med. (2019) 53(1):65–74. 10.1093/abm/kay017 29668841 PMC6301311

[92] Bhattarai M McDaniels B Jin Y Smedema SM. Pain and quality of life in persons with spinal cord injury: mediating effects of mindfulness, self-efficacy, social support, and functional independence. J Clin Psychol. (2024) 80(2):406–20. 10.1002/jclp.23616 37864835

[93] Toloui A Ramawad HA Gharin P Vaccaro AR Zarei H Hosseini M The role of exercise in the alleviation of neuropathic pain following traumatic spinal cord injuries: a systematic review and meta-analysis. Neurospine. (2023) 20(3):1073–87. 10.14245/ns.2346588.294 37798999 PMC10562228

[94] Santos LV Pereira ET Reguera-García MM Oliveira CEP de Moreira OC. Resistance training and muscle strength in people with spinal cord injury: a systematic review and meta-analysis. J Bodyw Mov Ther. (2022) 29:154–60. 10.1016/j.jbmt.2021.09.031 35248264

[95] Perry KN Nicholas MK Middleton J. Spinal cord injury-related pain in rehabilitation: a cross-sectional study of relationships with cognitions, mood and physical function. Eur J Pain. (2009) 13(5):511–7. 10.1016/j.ejpain.2008.06.003 18653364

[96] Kressler J Burns PA Betancourt L Nash MS. Circuit training and protein supplementation in persons with chronic tetraplegia. Med Sci Sports Exerc. (2014) 46(7):1277–84. 10.1249/MSS.0000000000000250 24389521

[97] van der Scheer JW Ginis M Ditor KA Goosey-Tolfrey DS Hicks VL West AL Effects of exercise on fitness and health of adults with spinal cord injury: a systematic review. Neurology. (2017) 89(7):736–45. 10.1212/WNL.0000000000004224 28733344

[98] Farrow M Nightingale TE Maher J McKay CD Thompson D Bilzon JLJ. Effect of exercise on cardiometabolic risk factors in adults with chronic spinal cord injury: a systematic review. Arch Phys Med Rehabil. (2020) 101(12):2177–205. 10.1016/j.apmr.2020.04.020 32445849

[99] Peters J Abou L Rice LA Dandeneau K Alluri A Salvador AF The effectiveness of vigorous training on cardiorespiratory fitness in persons with spinal cord injury: a systematic review and meta-analysis. Spinal Cord. (2021) 59(10):1035–44. 10.1038/s41393-021-00669-7 34274948

[100] Chiou SY Clarke E Lam C Harvey T Nightingale TE. Effects of arm-crank exercise on fitness and health in adults with chronic spinal cord injury: a systematic review. Front Physiol. (2022) 13:831372. 10.3389/fphys.2022.831372 35392374 PMC8982085

[101] Artacho-Cordón F Salinas-Asensio MDM Galiano-Castillo N Ocón-Hernández O Peinado FM Mundo-López A Effect of a multimodal supervised therapeutic exercise program on quality of life, pain, and lumbopelvic impairments in women with endometriosis unresponsive to conventional therapy: a randomized controlled trial. Arch Phys Med Rehabil. (2023) 104(11):1785–95. 10.1016/j.apmr.2023.06.020 37467936

[102] Boldt I Eriks-Hoogland I Brinkhof MWG de Bie R Joggi D von Elm E. Non-pharmacological interventions for chronic pain in people with spinal cord injury. Cochrane Database Syst Rev. (2014) 2014(11):CD009177. 10.1002/14651858.CD009177.pub2 25432061 PMC11329868

[103] Tabacof L Salazar SI Breyman E Nasr L Dewill S Aitken A Immersive virtual reality for chronic neuropathic pain after spinal cord injury: a pilot, randomized, controlled trial. Pain Rep. (2024) 9(6):e1173. 10.1097/PR9.0000000000001173 39391768 PMC11463206

[104] Maggio MG Bonanno M Calderone A Rizzo A Bulut N Bahramizadeh M Remapping body representation using virtual reality in chronic neuropathic pain: systematic review. J Med Internet Res. (2025) 27:e71074. 10.2196/71074 40341015 PMC12174876

[105] *National Spinal Cord Injury Statistical Center, Traumatic Spinal Cord Injury Facts and Figures at a Glance*. Birmingham, AL: University of Alabama at Birmingham (2025).

